# Assessing Chromium Contamination in Red Soil: Monitoring the Migration of Fractions and the Change of Related Microorganisms

**DOI:** 10.3390/ijerph17082835

**Published:** 2020-04-20

**Authors:** Siyuan Zhang, Xiaodong Hao, Jiahui Tang, Jin Hu, Yan Deng, Menglong Xu, Ping Zhu, Jiemeng Tao, Yili Liang, Huaqun Yin, Luhua Jiang, Xueduan Liu, Hongwei Liu

**Affiliations:** 1 School of Minerals Processing and Bioengineering, Central South University, Changsha 410083, China; siyuanzhang.bio@csu.edu.cn (S.Z.); haoxiaodongxyz@163.com (X.H.); jhtang@csu.edu.cn (J.T.); jinhu.bio@gmail.com (J.H.); dengyan@csu.edu.cn (Y.D.); xumenglong@csu.edu.cn (M.X.); zhuping199508@163.com (P.Z.); taojiemeng@csu.edu.cn (J.T.); liangyili@hotmail.com (Y.L.); yinhuaqun_cs@sina.com (H.Y.); jiangluhua@csu.edu.cn (L.J.); xueduanliu@yahoo.com (X.L.); 2 Key Laboratory of Biometallurgy of Ministry of Education, Changsha 410083, China

**Keywords:** Cr pollution assessment, fractions migration and occurrence, relative mobility, microbial indication, red soil

## Abstract

The improper stacking of chromium (Cr) slag poses a great threat to the environment and human health. The toxicity of Cr in soil is not only related to its total amount, but also to its fractions. A simulated experiment was conducted in laboratory to assess the environmental risk of Cr fractions migration and distribution in red soil. The results showed the content of acid-soluble and reducible Cr significantly decreased (*P* < 0.05) in top layer but increased in middle and substratum layers over time. This indicated that acid-soluble and reducible Cr migrated downward with time and the relative mobility of acid-soluble Cr (0.038 mg/kg·d·m) was higher than that of reducible Cr (0.028 mg/kg·d·m). Furthermore, correlation analysis between microbial community and chromium fraction showed the relative abundance of *Lysobacter*, *Flavihumibacter*, *Flavisolbacter,* and *Altererythrobacter* was significantly (*P* < 0.05) correlated with acid-soluble and reducible fractions. Thus, these microorganisms might be evaluators to assess the migration of acid-soluble and reducible fractions in red soil. In summary, this study provided a new comprehension on remediation of Cr-contaminated soil by monitoring the migration of acid-soluble and reducible fractions and the changes of related microbial groups.

## 1. Introduction

Industrial chromium (Cr) products have been widely applied in various important industrial applications, such as chemical, foundry, leather tanning, dyeing, and electroplating [1,2,3]. Cr slag abandoned by metallurgical and chemical industries contains a large amount of high-concentration Cr. Because of the lack of landfill sites and the expensive costs of Cr slag treatment, illegal disposing of the chromate plant has become a common problem in China [4]. The improper stacking of Cr slag under natural conditions generates soluble and migratable fractions of Cr, which may seep into the groundwater and result in the contamination of drinking water and agricultural soils [5,6,7]. The potential hazards of heavy metals in soils are dominated by their mobility and their ecotoxicological characteristics in solid–liquid phase [8]. Thus, decreasing of Cr bioavailability and hindering its fractions migration become the major concerns for remediation of Cr-contaminated soils [9].

It is well known that the environmental toxicity of Cr (VI) can be attributed to its water solubility and bioavailability [5,10]. The removal of Cr (VI) in soil environment is a great challenge due to its toxicity and mobility [11]. Several methods have been introduced for the detoxification of Cr-containing slag, such as hydro-based reduction, pyro-based reduction, and microbial reduction [12,13]. Due to the rapid diffusion of Cr (VI) in soil and aquatic environments, particular attention has been paid to the shifts in the soluble and exchangeable Cr fractions [14,15]. Many researches were conducted to change the different forms of Cr in the soil with the utilization of stabilizing and immobilizing agents, thereby reducing the bioavailability of Cr [16,17]. The hilly red soil, occupying an area of 1.131 million km^2^ and accounting for 11% of the total area of China, is an important resource in South China [18,19]. Excessive application of chemical fertilizers leads to the accumulation of Cr in red paddy soil, which affects the ecological environment and human health [20]. Therefore, it was vital to explore migration of Cr fractions in the red soil.

Soil microbes have been considered as sensitive indicators to heavy metal toxicity in the ecological environment [21]. The composition and structure of microbial communities have been well documented in Cr-contaminated soils [10,21,22]. Previous studies indicated that both archaeal and bacterial community composition of could shift greatly in the soil profile [23,24]. The rise of total Cr and hexavalent Cr concentration increased archaeal richness in soil, and *Proteobacteria*, *Firmicutes*, *Actinobacteria*, *Bacteroidetes,* and *Chloroflexi* were the major bacterial groups in Cr-contaminated soils [21,25,26]. In the study of bioremediation by microbial reduction, several bacteria have been shown to be capable of directly reducing Cr (VI) to Cr (III) under anaerobic or aerobic conditions [13,21,27], and the Cr fractions inevitably affected the microbial community structure, diversity, and abundance [28]. However, relationships between Cr fractions and microbial community on the detoxification of Cr were still unclear.

The migration and occurrence of Cr fractions will cause the change of environmental factors, and the shift of environmental factors inevitably affects the microbial community. Some studies indicated that the locally isolated strains showed higher tolerance to Cr as well as higher nitrogen fixation and higher phosphate and potassium solubilization capability [29]. Thus, it is necessary to search for Cr-resistant microorganisms with the capability of dissolving phosphorus and potassium, which can improve the soil fertility of red soil. Previous reports have shown a significant correlation between Cr migration and soil microorganisms [3,25,28]. Therefore, the current research explored the correlation between microbial communities, soil property, and Cr fractions, and assessed the toxicity of Cr fractions according to specific microbial groups.

Recently, BCR (European Community Bureau of Reference) three-step sequential extraction procedure, which divided the metals phase into acid extractable fraction, reducible fraction, oxidizable fraction, and residue fraction, has been an important method for monitoring soil fraction transformation [30,31,32]. Here, in order to investigate the migration and distribution of the four fractions of Cr and their corresponding impact on the microbial community, a simulation experiment in laboratory was carried out to (1) explore the migration and occurrence regularity of the four Cr fractions leached from the slag over time; (2) determine the vertical distribution and migration characteristics of the four Cr fractions in the soil profile; (3) evaluate the effects of the migration and occurrence of the four Cr fractions on soil microbial community and soil properties; (4) seek the correlation between Cr fractions and specific microbial groups.

## 2. Materials and Methods

### 2.1. Soil Sampling and Laboratory Simulation Experiment

**Figure 1:**
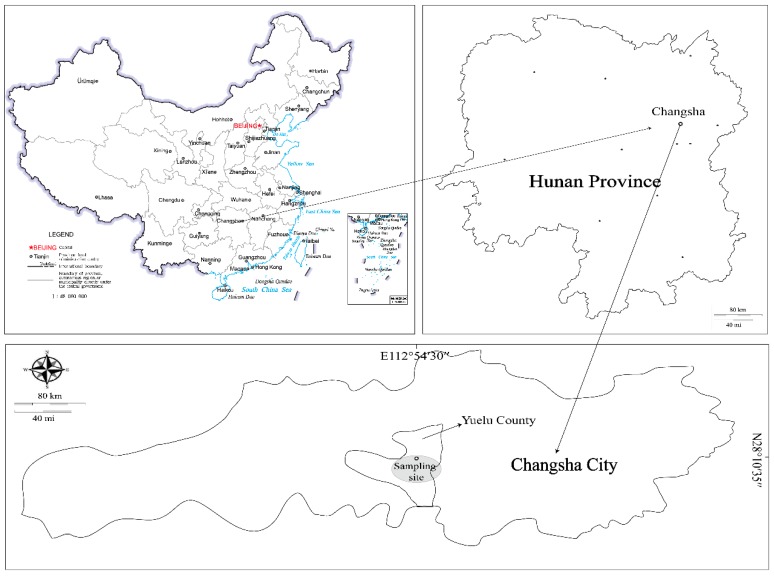
Map of sampling sites in the Yuelu County, China.

All the noncontaminated red soil samples were collected from the depth of 8–12 m of construction site in Yuelu County, Changsha City, Hunan Province, China (112°54′30″ E, 28°10′35″ N, Figure 1), then the soil samples from Changsha City were classified into three parts. The first part was frozen in liquid nitrogen immediately and stored in −80 °C freezer for molecular analysis. The second part was kept in the ice box and stored at 4 °C for soil physiochemical property analysis. The third part was air-dried at room temperature and then ground to pass through 0.84 mm sieves. Cr slags samples were gathered from an abandoned Cr salt factory located in Hunan Province, China (112°58′0″ E, 28°16′23″ N).

The laboratory simulation experiment was conducted on time and space scale to investigate the migration of Cr and its effects on microbial communities. The original soil (OS) was evenly mixed and placed in the six polymethyl methacrylate (PMMA) columns (diameter, 20 cm; height, 100 cm), and the PMMA columns were evenly distributed into four separate cells. Cr slag samples was evenly mixed and then ground to pass through 2 mm sieves. The height of red soil was accurately measured in the PMMA column. Then, Cr slag with height of 10 cm was evenly filled into the treatment group (the error was within 1 mm). The PMMA columns stacked with non-Cr slag (CK) and Cr slag (CR) were wrapped with foil to simulate the dark conditions. In addition, to simulate precipitation in Changsha City [33], the CK and CR groups were sprinkled with 205 mL water per week with spray kettle. Then, the red soil in CK and CR columns was sampled each month (30 d, 60 d, and 90 d). At each sampling time, the stacked Cr slag in PMMA column was completely removed. Then, the sampling point of cross section on PMMA column was marked every 20 cm. Based on the principle that the samples of each layer do not interfere with each other, three 20 cm thick red soil layers were taken. We took soil from different separate cells as three biological repeats of each layer of soil. The stacked Cr slag samples were defined as the Cr slag layer (G layer), then the first layer below it was defined as Top (T layer, 0–20 cm), the second layer was defined as Middle (M layer, 20–40 cm), and the third layer was defined as Substratum (S layer, 40–60 cm). Cr slags and each soil sample were classified into two parts, respectively, one part for soil property measurement and the other for community analysis.

Moreover, previous experiment and modeling research was concerned with the transfer of Cr (VI) from soil into surface runoff [34,35]. In this study, we calculated relative mobility of Cr fractions: r represented the relative mobility of Cr fractions (mg/kg·d·m), c1 was the concentration of the initial Cr fractions (mg/kg), c2 was the concentration of Cr fractions after migration with the time variation (mg/kg), t was the time of Cr fractions migration (d), and l was the soil depth (m).

(1) $$r=\frac{c1-c2}{c1\times \left(t\times l\right)}$$

### 2.2. Soil Property Measurement

Soil samples and Cr slag were digested by an acid mixture (HNO_3_, HF, and HClO_4_) on an electric heating plate (XJS20-42, Laboratory Instrument Equipment Co. Ltd., Tianjin, China) and total Cr (TCr) contents were measured by ICP-OES (Optima 5300DV, PerkinElmer, Shelton, Connecticut, USA). The four fractions of Cr in soil sample, including acid-soluble Cr (AC), reducible Cr (RED), oxidizable Cr (OX), and residual Cr (RES), were extracted by the three-step sequential extraction procedure as in our previous research [36]. Soil oxidation reduction potential (ORP) and pH were determined with the soil/water ratio of 5 g/25 mL by a pH meter (BPH-220, Bell Instrument Equipment Co. Ltd., Dalian, China). The soil organic matter (OM) was measured using the K_2_Cr_2_O_7_-H_2_SO_4_ oxidation–reduction colorimetric method. The total N (TN) was analyzed by Kjeldahl digestion. Nitrate nitrogen (NO_3_-N) and ammonium nitrogen (NH_3_-N) were analyzed using the calcium chloride method. Total K (TK) was determined by flame photometry after fusion of NaOH, and the available K (AK) was extracted by NH_4_OAc. The total P (TP) was detected by NaOH fusion followed by colorimetric assay. The available P (AP) was extracted with 0.5 mol·L^−1^ sodium bicarbonate (pH 8.5) and detected by colorimetry [37]. The properties of the soils are listed in Table S1.

### 2.3. DNA Extraction, Sequencing, and Data Preprocessing

Genomic DNA was extracted by Guangdong Magigene Biotechnology Co., Ltd., (Guangzhou, China) using commercial kits according to the manufacturer’s instructions. Sequencing libraries were constructed using NEB Next^®^ Ultra™ DNA Library Prep Kit for Illumina^®^ (New England Biolabs, MA, USA) following manufacturer’s guidance, and instruction code were added. The library was sequenced on an Illumina Hiseq X-ten platform, and 150 bp paired-end reads were generated. Trimming of reads with quality scores less than 20 (Q20) was performed by Btrim program [38]. Forward and reverse reads of same sequence were merged by FLASH program [39]. After trimming, the FASTQ data was converted to FASTA format. Then, sequences with 97% identity to the same operational taxonomic unit (OTU) were clustered using UPARSE. [40]. Taxonomic assignment was performed using RDP classifier (http://rdp.cme.msu.edu/classifier/classifier.jsp).

Alpha-diversity (Pielou’s evenness, Shannon index, and observed_species) of soil microbial communities was analyzed by the Institute for Environmental Genomics (IEG), University of Oklahoma website (http://ieg.ou.edu/). The Venn graph was drawn using “Venn Diagram” Package. Principal co-ordinates analysis (PCoA) for comparing different group sites was conducted on the R statistical platform with the “Vegan” Package. Redundancy analysis (RDA) for assessing the relationship between microbial community structures and environmental factor was also performed on R with the “Vegan” Package. Minitab 17.0 software (Minitab Inc.) was used to perform a Spearman correlation analysis of AC, RED, OX, and RES fractions and the abundance of top 10 microbial genera. The correlation coefficient was calculated and shown in the heat map.

Statistical analyses were performed using Minitab 17.0 software (Minitab Inc., Pennsylvania State University, Pennsylvania, USA). One-way ANOVA analyses, followed by Tukey’s test, were carried out to identify the difference in parameters (e.g., soil physio chemical properties and diversity indexes) between each sample. The least significant difference test (LSD) was used to identify significant (*P* < 0.05) differences between means. We have used Shapiro–Wilk method to test the normal distribution of the original data. Pearson correlation analyses were used to identify quantitative relationship between microbial abundance and soil properties contents. Generally, a *P*-value below 0.05 was considered significant. Sequencing data had been submitted to NCBI sequence read archive (SRA) database, and the accession number was PRJNA579018.

## 3. Results and Discussion

### 3.1. Migration and Occurrence of Cr Fractions in Red Soil with Time and Space Distribution

As shown in Table S2, compared with the non-Cr stacked control group (CK), the Cr content of the four fractions in the treatment group (CR) increased significantly (*P* < 0.05) in soil. Additionally, compared with the CK, the RES fraction in the CR group decreased from 58.2% to 15.1%, while the AC and RED fractions increased from 6.3% to 15.9% and 8.5% to 42.3%, respectively. Therefore, there was downward migration of Cr with rainfall leaching, and the proportion and contents of Cr in four fractions had significantly altered.

**Figure 2:**
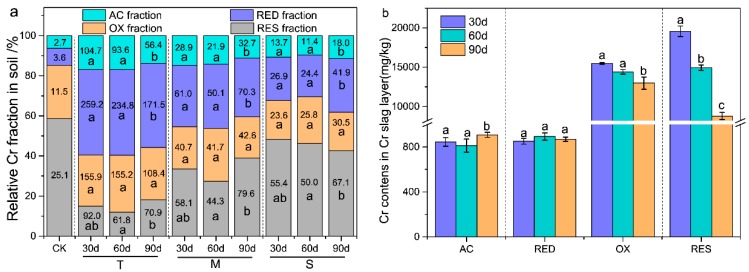
(**a**) Relative percentage of Cr fraction in soil, and the numbers in the columnar section represent the Cr content. (**b**) The contents of each Cr fraction in the G layer (Cr slag layer) soils. Data are presented as means ± SD (n = 3). Different lowercase letters above the bars indicate significant difference (*P* < 0.05, LSD) among different groups. CK: control group; T: top layer; M: middle layer; S: substratum layer; AC: acid-soluble Cr; RED: reducible Cr; OX: oxidizable Cr; RES: residual Cr; LSD: least significant difference

As shown in Figure 2a, the content of AC and RED fractions significantly decreased in T layer but increased in M and S layers over time (*P* < 0.05). The results showed that AC and RED fractions could migrate downward with time and the relative mobility of AC fraction (0.038 mg/kg·d·m) was higher than RED fraction (0.028 mg/kg·d·m). This was in agreement with the previous study that showed metals in exchangeable fraction were predominant in the spiked soils after 3 h of incubation [41]. The G layer had a high content of OX and RES fractions, which were mostly in a relatively stable state during the long-term stacking of Cr slag (Figure 2b). Meanwhile, the four fractions of G layer could be transformed with time. In 60 to 90 days, the AC fraction of G layer increased, while the OX and RES fractions decreased. The results showed that the OX and RES fractions of G layer were transformed into AC fraction, and migrated to the next layer of soil subsequently. Afterwards, the water-soluble Cr fraction was transformed into the four Cr fractions in a specific proportion. As the soil was saturated with water during the rainfall simulation, AC and RED fractions eventually migrated downward. Hence, for these four Cr fractions, AC and RED fractions could migrate downward into groundwater, and thereby caused greatly environmental risks.

Water-soluble Cr fraction was transformed into these four Cr fractions in soil by rainfall leaching of the Cr slag. The maximum Cr content of these four fractions was RED fraction, followed by OX, RES, and AC fractions, and the four fractions distribution was related to soil physical and chemical properties. Red soil was rich in Fe, Mn, and Al, and the RED fraction of metals accounted for a higher proportion [42]. A reducing reagent (E0 = −1.87 V and pH 2.0) of hydroxylamine hydrochloride was used to dissolve this metal fraction, and the Cr of strong reduction ability was combined with hydrous oxides of Fe, Mn, and Al to form a relatively stable RED fraction in red soil. The OX fraction was mainly incorporated in complex polymeric substance (e.g., humus) and other organic products such as proteins, carbohydrates, and fats. The organic substance and desorption metals tended to be degraded under a peroxidation condition by the oxidizing reagents such as H_2_O_2_ (E0 = 1.8 V) or NaClO (E0 = 0.9 V). As shown in a previous study [43], AC fraction of metals was mostly presented as co-precipitated with carbonate minerals, which were sensitive to pH changes, and metal release was achieved through the dissolution of solid materials at a condition of pH 5. In this study, soil pH 7.38 was increased to pH 7.97 (Table S1) with the addition of the water-soluble Cr (pH 8.2). Therefore, the contents of AC fraction were lower than that of other fractions in soil. Metals were coated into the crystalline lattice of silicate, which was the primary and secondary mineral constituting the RES fraction of metals. The metals release was achieved by the digestion with strong acids such as HF, HClO_4,_ and HNO_3_. The result indicated that soil properties were the key factors affecting the occurrence of Cr fractions in the red soil. Meanwhile, some studies have proved that different soil properties could significantly affect the occurrence of Cr fraction [28]. However, there was no significant difference (*P >* 0.05) in the contents of RES fraction with time and space distribution, and some researches showed that Cr in the RES fraction was not easily released in nature conditions, so the proportion of RES fraction heavy metals remained basically stable [41,44,45]. Compared with other types of soil, the RES fraction content in red soil was lower and difficult to transform, thus Cr pollution in red soil easily caused greater environmental toxicity.

In red soil, AC and RED fractions migrated downward with time, and soil properties affected the occurrence of Cr fractions. Meanwhile, it was difficult to transform the RES fraction into other fractions in a short time. Thus, it was important to evaluate the toxicity of Cr in red soil by the migration and occurrence of Cr fractions.

### 3.2. Relationships between Four Fractions of Cr and Soil Properties

Changes in soil properties are shown in Table S1. The result showed that the downward migration of AC and RED fractions changed soil chemical properties. In Lu’s studies [41], soil pH and organic matter played an important role in metal fraction distribution patterns. However, our study indicated that there were other factors affecting the fractions transformation of exogenous Cr. Relationships between four fractions of Cr and soil properties were also shown by correspondence analysis (CA) in the Table 1. The AC fraction and the RED fraction were positively related to OM, pH, and NH_3_-N, while NO_3_-N showed negative relationships with AC fraction and RED fraction. The OX fraction had positive relations to OM (r = 1, *P* = 0.02). The RES fraction had negative relations to AK (r = −0.995, *P* = 0.043). Therefore, this result indicated that the OM, AK, NO_3_-N, NH_3_-N, and pH of soil properties were closely related to the Cr fractions.

Additionally, the OM, NH_3_-N, and pH were positively related to the AC and RED fractions (Table 1). It has been reported that the soil pH, calcium carbonate, and organic matter content were the key factors controlling the distribution of Cr fractions [45]. The pH reduction during nitrification was in agreement with those studies [46,47,48]. This research demonstrated that the binding of Cr was influenced by pH and might also be influenced by ammonium and organic matter. Taken together, the AC and RED fractions of Cr migrated downward with time. Meanwhile, the two fractions were positively correlated with OM, NH_3_-N and pH, respectively. Previous studies have shown that exogenous heavy metals were quickly adsorbed on the surface of soil particles to form exchangeable heavy metals, which can be further converted to other tightly bound fractions over time, and the four fractions of Cr may be strongly influenced by changing soil conditions [41,48]. In addition, N, P, and K (inorganic fertilizer) could be applied to lessen the toxicity of Cr in soil and to maintain the strength of physiological growth of plants [49]. Therefore, the effect of Cr fractions migration on soil fertility was evaluated by measuring the shifts of N, P, and K elements, and the OM, NH_3_-N, and pH of soil properties were positively related to the AC and RED fractions.

### 3.3. Correlation between the Microbial Community and Environmental Factor

**Figure 3:**
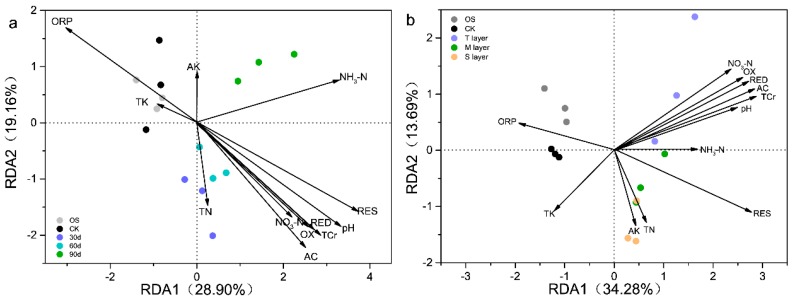
The ordination plot from redundancy analysis (RDA) showing the relationship between the microbial community structures and environmental variables. (**a**) With the time variation; RDA Axis 1 explained 28.90% and RDA Axis 2 explained 19.16% of the total variance; (**b**) with the depth change; RDA Axis 1 explained 34.28% and RDA Axis 2 explained 13.69% of the total variance. Each point represents the individual microbial community in soils. Arrow direction indicates the correlation among soil properties; arrow length indicates the strength of the correlation. OS: original soil; ORP: oxidation reduction potential; TN: total N; NH_3_-N: ammonium nitrogen; NO_3_-N: nitrate nitrogen; TK: total K; AK: available K; T Cr: total Cr.

The ordination plot from redundancy analysis (RDA) was used to reveal the relationship between environmental factors, Cr fraction migration, and microbial community. As shown in Figure 3a, the NO_3_-N, pH, ORP, T Cr, AC, RED, OX, and RES fractions were significantly correlated to the bacterial composition (permutational multivariate analysis of variance, *P* < 0.05) with the time variation. The projective distance between the microbial composition at day 90 and NH_3_-N was shorter than that with other environment factors. Previous research showed that abundance of indigenous microorganisms in soil was affected by the combination of these contaminants (nitrogen, Cr, and high salinity) [28]. As we all know, the direction indicated by the arrow represents the index of community analysis. The angle between the arrow and the sorting axis indicates correlation, and the acute angle indicates positive correlation. With the downward migration of AC and RED fractions, NH_3_-N had a significant positive correlation with the composition and abundance of microbial communities. The Mantel test (r = 0.428, *P* = 0.003) was performed to further prove that NH_3_-N was not only positively correlated with the AC and RED fractions (Table 1), but also used for the indicator of specific microbial strains (Table S3).

As shown in Figure 3b, the NO_3_-N, pH, AC, RED, OX fractions and T Cr were significantly related to the bacterial composition in T layer. In addition, the microbial composition of the M and S groups projected to a shorter distance of the environment factor RES fraction than other fractions. Compared with the other three fractions, the correlation between RES fraction and soil microbial composition was significantly different. Mental test proved that AC (r = 0.571, *P* = 0.001) and RED (r = 0.496, *P* = 0.001) fractions were significantly related to the microbial composition of T layer soil, yet the RES fraction (r = 0.674, *P* = 0.001) was correlated to the bacterial community in M and S layers (Table S4). Therefore, the RES fraction was a stable fraction and its correlation with microbial composition was significantly different from that of the other three fractions in different depths. This further indicated that spatial distribution not only affected the occurrence of Cr fractions, such as the contents of AC, RED, and OX fractions, which were decreased with the increase of depth (Table S2), but also altered the correlation between Cr fractions and microbial communities in the soil profile.

Thus, the content of NH_3_-N was not only positively correlated with the AC and RED fractions, but also affected the microbial composition in Cr-contaminated soil. RES fraction was a stable fraction and the correlation with microbial composition was significantly different from that of the other three fractions.

### 3.4. Soil Microbial Community Composition and Diversity

**Figure 4:**
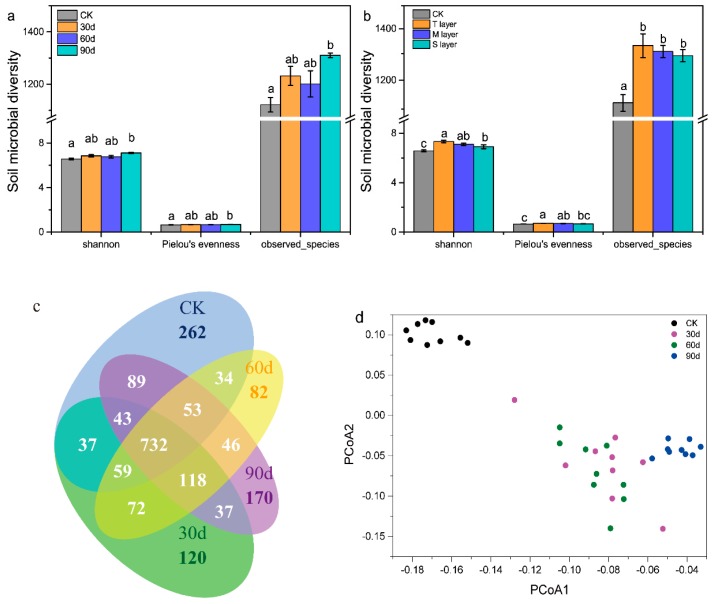
(**a**) Alpha-diversity indexes in soils with the time variation; (**b**) alpha-diversity indexes in soils with the depth change. Venn diagram (**c**) and principal co-ordinates analysis (PCoA) (**d**) of microbial community in soils with the time variation. Each point represents the individual microbial community in soils. Data are presented as means ± SD (n = 3). Different lowercase letters above the bars indicate significant difference (*P* < 0.05, LSD) among different groups.

A total of 49,854,345 high-quality 16s rRNA gene reads were obtained for all 66 samples, which can be clustered into 5797 OTUs. As shown in Figure 4a, from 0 to 60 days, there were no significant (*P >* 0.05) differences in soil microbial alpha-diversity measures (including Shannon, Pielou’s evenness, and observed species) between the CK and CR groups. As shown in Figure 4b, with the Cr fractions migration, there was no significant difference (*P >* 0.05) in microbial alpha-diversity among different layers of soil at the same time. This was in agreement with the previous study that showed there was no clear trend for the shift of bacterial alpha-diversity with soil depth [25]. Hence, there was no significant difference in microbial alpha-diversity in shallow soil (0–60 cm) with spatial distribution. The microbial diversity of the whole soil was analyzed with time distribution. During 60 to 90 days, compared with the CK group, the microbial alpha-diversity of the CR group obviously increased (*P* < 0.05). The results were in agreement with our conclusion, that microbial alpha-diversity increased when AC and RED fractions reduced from 60 to 90 days.

The Venn diagram showed that there were 688 shared OTU among the five types of soil samples. As shown in Figure 4c, the unique OTU in the CR group was higher than that in the CK group. From 60 to 90 days, the unique OTU in the CR group increased significantly. This study suggested that the AC and RED fractions in soil migrated downward and flowed out from the PMMA column, which reduced the toxicity to native microorganisms and improved microbial diversity. Therefore, in the present study, AC and RED fractions migration displayed evident influence on soil microbial diversity, which was consistent with the results reported in the literatures [21,22]. For beta-diversity analysis of soil microbial community, PCoA was conducted based on the Bray–Curtis distance of OTU matrix with time (Figure 4d). PCoA ordination plot showed that bacterial communities were not clustered together in soil profile (Figure S1), which was in accordance with the results of microbial alpha-diversity and dissimilarity analyses (Table S5). In previous reports, the WPGMA (weighted pair group method with averaging) clustering illustrated that bacterial communities at 0.5–2.5 m soil depths were clustered together [25]. Thus, PCoA analysis of different time distribution showed that clusters of bacterial communities were separated by 90 days group and other sampling times, and the dissimilarity results further confirmed this result (Table S6). In our previous study, the AC and RED fractions migrated downward, which reduced the toxic effect on the microbial community, and caused differences in microbial community among samples at different times. Here, with the successive leaching of rainfall, the contents of AC and RED fractions decreased in 60–90 days, and then the diversity of soil microorganisms increased significantly.

**Figure 5:**
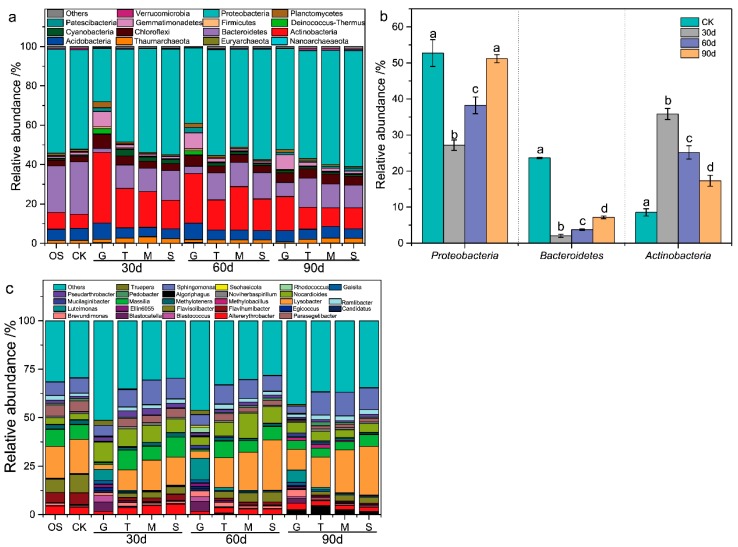
Relative abundance of microbial phyla in different soil samples. (**a**) An overall view, and (**b**) the most abundant three phyla. Others included *Acidobacteria, Chloroflexi, Deinococcus-Thermus, Gemmatimonadetes, Patescibacteria,* and *Planctomycetes*; (**c**) genus level. Data are presented as means ± SD (n = 3). Different lowercase letters above the bars indicate significant difference (*P* < 0.05, LSD) among different groups. G: Cr slag layer.

The variation of bacterial community structure was monitored during the Cr fractions migration process, and as shown in Figure 5a, the dominant microbial phyla (>2%) collected from the bacterial consortium were Proteobacteria, Bacteroidetes, and Actinobacteria in the CK and CR groups, which was consistent with the previous research of the bacterial communities in the Cr-contaminated soils [10,21]. Additionally, phyla of Proteobacteria and Bacteroidetes showed a significant increase (27.2%–51.2% and 2.1%–7.1%, *P* < 0.05), while Actinobacteria significantly reduced (35.8%–17.3%, *P* < 0.05) in G layer over time (Figure 5b). In this study, the TCr content of G layer decreased with time variation (Table S1). Thus, the reduction of TCr content was negatively correlated with the relative abundance of Proteobacteria and Bacteroidetes, and was positively correlated with the relative abundance of Actinobacteria. The results were consistent with the former reports that Actinobacteria had great resistance to Cr-contaminated soil [10,22,25]. Obviously, the downward migration of Cr fractions altered the relative abundance of microbial community in phyla level.

The genus-level characterization further demonstrated the variations in relative abundance of bacterial community with the migration of Cr fractions. After filtering those genera whose functions were unclear or whose abundance was negligible, 32 genera remained, as displayed in Figure 5c. Compared with the CK group, the relative abundance of *Flavihumibacter, Flavisolibacter, Lysobacter, Methylotenera, Massilia, Parasegetibacter,* and *Sphingomonas* was significantly reduced in G layer and T layer. Thus, the Cr fractions migration and occurrence showed a significant toxic effect for these genera.

With the downward migration of the AC and RED fractions, the relative abundance and diversity of microbial phyla and genus in the Cr-contaminated soil profile were significantly different from those of the uncontaminated soils. Therefore, seeking for the correlation between specific microbial genera and Cr fractions could provide useful information for remediation of Cr-contaminated soil.

### 3.5. Correlation between the Microbial Communities and Migration of Four Cr Fractions

**Figure 6:**
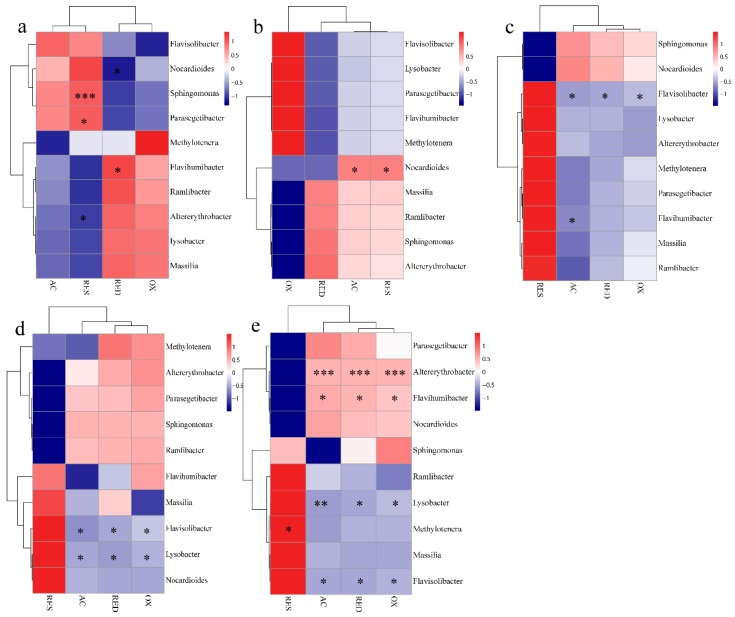
Correlation heat map of the top ten genera and four fractions of Cr in original soil (**a**), CK (**b**), 30 d (**c**), 60 d (**d**), 90 d (**e**). X and Y axis are four fractions of Cr and genera, respectively. R in different colors to show; the right side of the legend is the color range of different r values. The value of *P* < 0.05 is marked with “*”, *P* < 0.01 is marked with “**”, and *P* < 0.001 is marked with “***”.

There was no significant difference in microbial diversity in shallow soil (0–60 cm) with spatial distribution (Figure 4). The correlation between microbial diversity and Cr fractions in the whole soil is shown in Figure 6. The relationships between four fractions of Cr and the top ten microbial genera were changed in Cr-contaminated soil. With the time change, *Flavisolibacter* was obviously negatively related to AC, RED, and OX fractions (Figure 6c–e). *Flavisolibacter*, Gram-negative, chemoheterotrophic, nonmotile, aerobic, and positioned phylogenetically within the phylum Bacteroidetes, was resistant to Cr and was found as a dominant genus in the Cr-polluted soil [50,51]. As shown in Figure 5c, the relative abundance of *Flavisolbacter* in the CR group was significantly lower than that in the CK group. Additionally, *Flavihumibacter* was negatively correlated with AC (r = −0.673, *P* = 0.047) fraction in the 30 d group, and positively correlated with AC (r = 0.747, *P* = 0.021) fraction in the 90 d group. As shown in Figure 5c, the relative abundance of *Flavihumibacter* decreased with the increase of Cr content, so AC fraction had a more significant negative effect on *Flavihumibacter*. In the 60 d and 90 d groups, *Lysobacter* was negatively correlated with AC and RED fractions. That was consistent with our previous result (Figure 5c). With the downward migration of AC and RED fractions, the content of TCr decreased while the relative abundance of *Lysobacter* increased. Some studies have described the resistance of *Lysobacter* spp. to Cr [52,53]. Thus, the migration of AC and RED fractions affected microbial communities, and the relative abundance of *Lysobacter* and *Flavisolbacter* was significantly and negatively correlated with RED fraction, and the relative abundance of *Lysobacter*, *Flavihumibacter,* and *Flavisolbacter* was significantly and negatively correlated with AC fraction. Previous studies have shown that some microbial strains were inhibited by the presence of heavy metals, and some genera (such as *Lysobacter*, *Ramlibacter*, *Flavihumibacter,* and *Flavisolibacter*) had been identified in the analyzed samples [51,54,55,56]. In the 90 d group, *Altererythrobacter* was positively correlated with AC (r = 0.932, *P* = 0) and RED (r = 0.916, *P* = 0.001) fractions. In our previous results, *Altererythrobacter* positioned phylogenetically within the phylum Proteobacteria, and its relative abundance was positively correlated with the decreases of AC and RED contents (Figure 2a), indicating *Altererithrobacter* had obvious tolerance in Cr-contaminated soil. Previous study also showed that *Altererythrobacte* had obvious resistance to heavy metals, and it may have the potential for biotechnological applications in bioremediation of heavy metal-contaminated environment [57].

## 4. Conclusions

In summary, this work explored Cr migration and microbial response in the red soil. AC and RED fractions migrated downward with the time, and the relative mobility of AC fraction (0.038 mg/kg·d·m) was higher than that of RED fraction (0.028 mg/kg·d·m). For soil properties, the AC and RED fractions were positively correlated with OM, NH_3_-N, and pH, and for microbial community, the relative abundance of *Lysobacter*, *Flavihumibacter*, *Flavisolbacter,* and *Altererythrobacter* was significantly correlated with AC and RED fractions. Therefore, the dual impact factors of soil properties and microorganisms monitored the migration of AC and RED fractions. This provided useful information and new insights for using microbial groups to assess the environmental toxicity in the future.

## Figures and Tables

**Table 1:** Pearson correlation coefficients between Cr of four fractions and soil properties with the time variation.

Time	Fractions	Properties					
		OM	TP	AK	NH_3_-N	NO_3_-N	pH
30 d	AC	1 **	n.s.	n.s.	0997 *	−0997 *	n.s.
	RED	1 **	n.s.	n.s.	0995 *	−0995 *	n.s.
	OX	1 *	n.s.	n.s.	n.s.	n.s.	n.s.
	RES	n.s.	n.s.	0.995 *	n.s.	n.s.	n.s.
60 d	AC	099 *	0.991 *	n.s.	1 ***	n.s.	n.s.
	RED	0991*	0.993 *	n.s.	1 **	n.s.	n.s.
	OX	0991*	0.995 *	n.s.	1 **	n.s.	n.s.
	RES	n.s.	n.s.	n.s.	n.s.	n.s.	n.s.
90 d	AC	n.s.	n.s.	n.s.	n.s.	n.s.	0.992 *
	RED	n.s.	n.s.	n.s.	n.s.	n.s.	0.993 *
	OX	n.s.	n.s.	n.s.	n.s.	n.s.	n.s.
	RES	n.s.	n.s.	n.s.	n.s.	n.s.	n.s.

Note: OM: soil organic matter; TP: total P; NH_3_-N: ammonium nitrogen; NO_3_-N: nitrate nitrogen; AK: available K; AC: acid-soluble Cr; RED: reducible Cr; OX: oxidizable Cr; RES: residual Cr. The no significant correlation is marked with “n.s.”, the value of *P* < 0.05 is marked with “*”, *P* < 0.01 is marked with “**”, and *P* < 0.001 is marked with “***”.

## Supplementary Materials

The following are available online at https://www.mdpi.com/1660-4601/17/8/2835/s1, Figure S1: Principal co-ordinates analysis (PCoA) of microbial community in soils with the space variation, Table S1: Soil physicochemical properties (means ± SD, n = 3) with time and space distribution, Table S2: soil Cr fractions with time and space distribution, Tables S3 and S4: mantel test of the relationship between the bacterial community structure and soil characteristics with the time and space variations, Tables S5 and S6: the dissimilarity analysis among different depth and time groups.
