# Supplementary material for: Assessing Chromium Contamination in Red Soil: Monitoring the Migration of Fractions and the Change of Related Microorganisms
Source: Int J Environ Res Public Health. 2020 Apr 20;17(8):2835. doi: 10.3390/ijerph17082835 (PMC7215348; doi:10.3390/ijerph17082835)
Supplement: Supplementary file 1 [file ijerph-17-02835-s001.pdf]

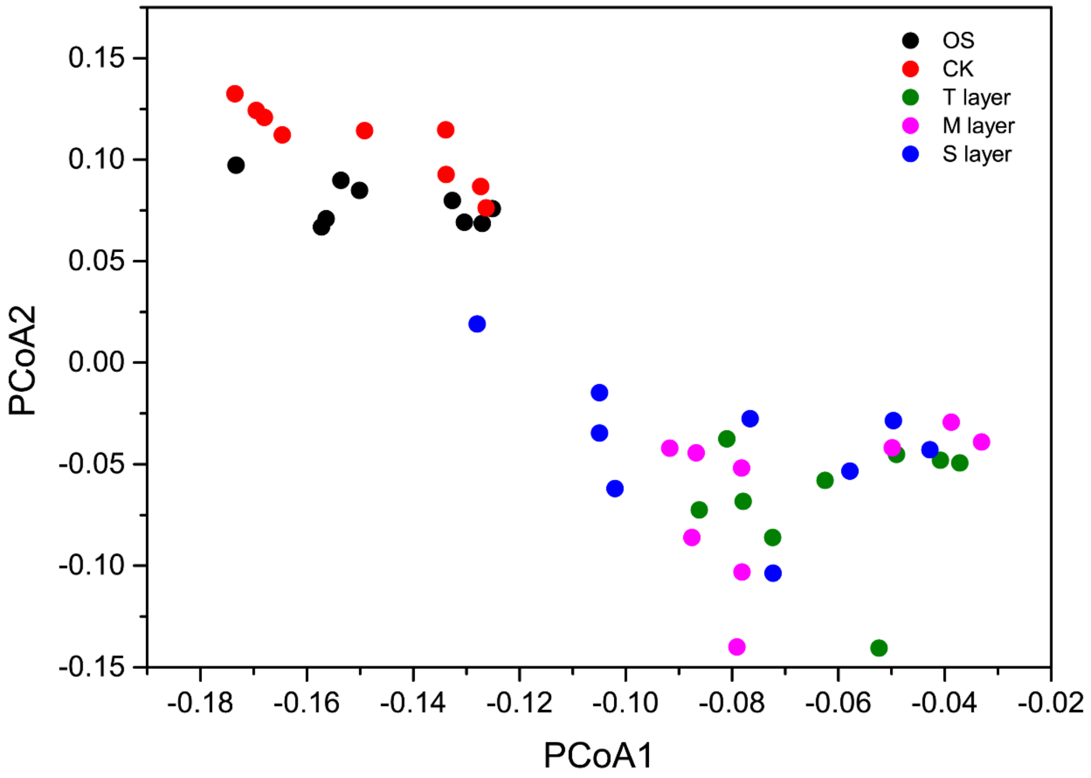

Figure S1. Principal co-ordinates analysis (PCoA) of microbial community in soils with the space variation. Each point represents the individual microbial community in soils OS: original soil.

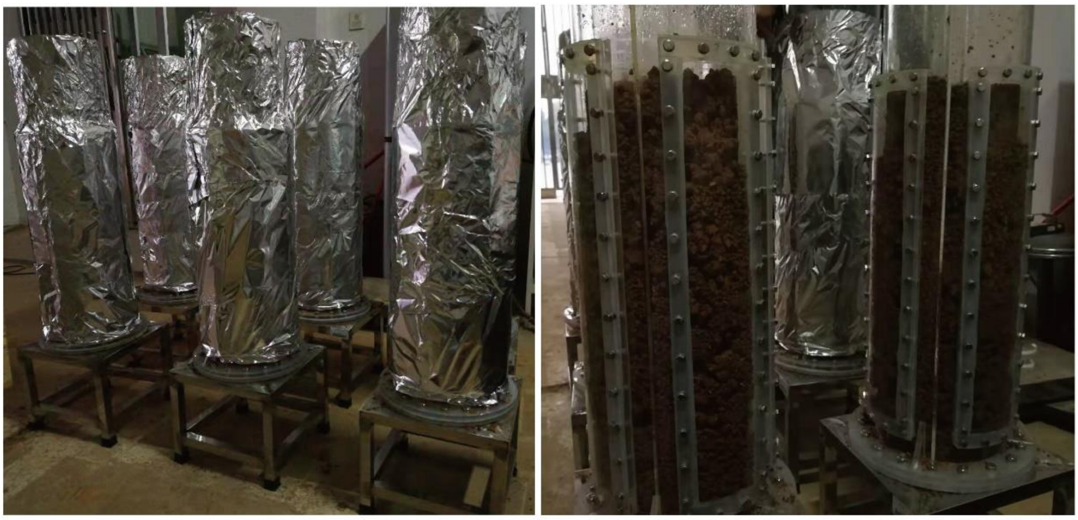

Figure S2. Polymethyl methacrylate (PMMA) columns.

Table S1 Soil physicochemical properties (means ± SD, n = 3) with time and space distribution

Properties	OS (0day)	layer	CK	Time		
				30d	60d	90d
OM (%)	0.16±0.08	G	0	0.4 ±0.2a	0.5 ±0.1a	0.16±0.3a
		T	0.7±0.13a	0.98±0.25b	0.86±0.33ab	0.17±0.08c
		M	0.4±0.03a	0.39±0.05a	0.15±0.08b	0.13±0.04b
		S	0.4±0.11a	0.27±0.03a	0.16±0.12a	0.26±0.13b
TN (mg/Kg)	369.50 ±44.31	G	0	1467.7±542.1b	2875.3±643.2a	1715.5±343.2b
		T	421.4±16.7c	575.5±58.7b	502.1±46.9b	651.6±22.3a
		M	498.1±51.7a	444.1±397.0a	480.5±38.5a	476.0±28.6a
		S	538.1±15.6b	558.8±19.9b	1605.7±235.4a	369.6±27.6c
TP (mg/Kg)	74.52 ±5.31	G	0	91.5±22.3c	263.5 ±33.3a	168.8 ±42.3b
		T	109.2±2.0b	109.0 ±2.3b	93.0±4.5c	153.3±4.8a
		M	126.4±3.5b	325.6±12.0a	127.3±4.8b	104.0±3.2c
		S	142.8±4.3a	121.5±9.6b	110±8.4b	87±6.3c
AP (mg/Kg)	67.16 ±7.31	G	0	22.36±5.3a	28.42 ±4.3a	28.54 ±0.3a
		T	24.2±4.4a	25.56±5.5a	30.3±4.8a	28.59±3.3a
		M	24.0±5.4a	23.18±6.3a	29.1±7.3a	27.2±2.3a
		S	26.7±0.9a	24.2±6.2a	25.7±8.1a	28.55±5.2a
NH_3-N (mg/Kg)	4.45±0.66	G	0	9.66 ±0.3b	9.5±2.9b	13.18±0.6a
		T	5.2±1.5b	8.4±0.6a	8.6±0.7a	8.8±1.6a
		M	5.2±1.4b	5.7±0.2b	5.9±0.9b	16.6±3.6a
		S	4.6±0.9c	4.8±0.3b	5.5±1.6b	16.1±4.8a
NO_3-N (mg/Kg)	25.27 ±3.65	G	0	180.5 ±3.3a	141.33±4.5b	97.94 ±0.4c
		T	26.2±2.1c	36.6±2.2a	39.2±3.8a	31.6±1.1b
		M	27.0±3.1a	28.1±3.8a	28.1±2.1a	29.4±4.2a
		S	24.6±3.4a	25.4±2.8a	29.2±4.9a	27.4±3.6a
TK(g/Kg)	18.17±2.56	G	0	11.16 ±0.3b	11.29 ±3.8ab	15.31 ±0.6a
		T	14.2±2.2a	13.9±4.2a	10.2±3.1a	13.5±1.2a
		M	13.1±0.5a	13.3±0.2a	15.3±3.2a	13.2±1.9a
		S	14.4±0.5a	13.5±1.1a	14.5±2.5a	12.9±3.4a
AK (mg/Kg)	105.67±4.48	G	0	43.13±6.3a	32.77 ±4.31b	63.14 ±5.9c
		T	112.5±3.0a	105.3±5.8ab	101.3±4.1b	114.6±2.8a
		M	112.4±9.5a	113.9±4.9a	103.8±2.8b	113.2±3.3a
		S	107.7±7.4a	113.5±6.6a	112.2±2.5a	111.3±3.8a
pH	7.38±0.47	G	0	8.2±0.2a	8.11±0.3a	8.15±0.2a
		T	7.6±0.07b	7.9±0.04a	7.9±0.06a	7.9±0.07a
		M	7.6±0.06a	7.7±0.07a	7.7±0.04a	7.7±0.03a
		S	7.5±0.03b	7.7±0.02a	7.5±0.1b	7.6±0.09ab
ORP	206.73 ±12.56	G	0	202.3 ±3.6b	205.6±3.6ab	211.35 ±10.6a
		T	235.7±6.7a	177.4±9.6c	199.4±8.6ab	188.9±11.6b
		M	225.0±10.7a	207.1±12.1ab	198.5±3.6 b	195.3±5.4b
		S	219.2±8.0a	197.1±7.7b	199.1±4.1b	199±2.1b

T Cr (mg/Kg)	48.69 ±2.35	G	0	36728.2±448.6a	31050±667.1b	23527.9±745.2c
		T	42.8±3.9d	611.7±18.4a	545.3±23.5b	407.1±13.9c
		M	43.6±1.2c	188.6±33.5ab	157.9±12.8b	205.2±22.3a
		S	41.6±3.0c	119.6±4.3b	111.5±3.2b	157.4±8.8a

Different lowercase letters in same row indicated significant difference ($P < 0.05$, LSD) among different groups. ORP: oxidation reduction potential; OM: soil organic matter; TN: total N; TP: total P; AP: available P; NH_3-N: ammonium nitrogen; NO_3-N: Nitrate nitrogen; TK: total K; AK: available K; T Cr: total Cr; OS: original soil; CK: control group; G: Cr slag layer; T: top layer; M: middle layer; S: substratum layer.

Table S2 Soil Cr fractions (means ± SD, n = 3) with time and space distribution.

Cr fractions	OS (0d)	layer	CK	Time		
				30d	60d	90d
AC (mg/kg)	2.6±0.39	G	0	845.1±38.5b	812.5±58.2b	908±22.9a
		T	2.76±0.04c	104.72±8.87a	93.6±13.25a	56.36±8.23b
		M	2.64±0.02c	28.85±5.23b	21.92±2.31b	32.74±2.851a
		S	2.64±0.02c	13.65±3.18b	11.36±3.27b	17.97±2.12a
RED (mg/kg)	3.8±0.27	G	0	850.5±24.8a	893.1±33.1a	868.5±18.9a
		T	3.72±0.04c	259.2±13.33a	234.81±12.14a	171.45±10.51b
		M	3.6±0.23c	61.04±5.85b	50.09±4.31b	70.26±7.30a
		S	3.64±0.40c	26.94±3.83b	24.4±4.55b	41.90±3.65a
OX (mg/kg)	6.9±1.15	G	0	15470±112.3a	14410±288.87a	12965±778.87b
		T	11.55±0.10b	155.86±23.93a	155.16±22.64a	108.38±20.00a
		M	11.25±0.29b	40.71±12.32a	41.66±7.50a	42.6±7.67a
		S	11.75±0.12b	23.63±8.04a	25.81±6.16a	30.45±5.80a
RES (mg/kg)	35.5±3.42	G	0	19562.6±677.5a	14934.3±338.8b	8786.4±446.5c
		T	26.01±1.57c	92.00±18.87ab	61.81±11.04a	70.86±5.84b
		M	25.17±0.92c	58.06±10.59ab	44.27±8.26b	79.59±7.97a
		S	24.65±0.45c	55.42±4.46ab	50.02±5.35b	67.08±10.51a

Different lowercase letters in same row indicated significant difference ($P<0.05$, LSD) among different groups. AC: acid-soluble Cr; RED: reducible Cr; OX: oxidizable Cr; RES: residual Cr.

Table S3 Mantel test of the relationship between the bacterial community structure and soil characteristics with the time variation

Envs	r	p
**TN**	-0.122	0.754
**NO3**	0.037	0.379
**NH3**	0.428	**0.003**
**TK**	-0.193	0.929
**AK**	-0.125	0.882
**pH**	0.418	**0.001**
**ORP**	0.367	**0.009**
**T Cr**	0.359	**0.008**
**AC**	0.293	**0.010**
**RED**	0.316	**0.014**
**OX**	0.289	**0.012**
**RES**	0.634	**0.001**

Table S4 Mantel test of the relationship between the bacterial community structure and soil characteristics with the depth change

Envs	r	p
**TN**	0.699	0.599
**NH3**	0.001	0.415
**NO3**	0.325	**0.006**
**TK**	0.173	0.113
**AK**	0.866	0.177
**pH**	0.404	**0.003**
**ORP**	0.218	0.051
**T Cr**	0.586	**0.001**
**AC**	0.571	**0.001**
**RED**	0.496	**0.001**
**OX**	0.444	**0.001**
**RES**	0.674	**0.001**

Table S5 The dissimilarity analysis among different depth groups

ANOSIM	OS	CK	T	M	S
**OS**	0	0.001	0.001	0.001	0.001
**CK**	0.874	0	0.001	0.001	0.001
**T**	0.86	0.841	0	0.099	0.054
**M**	0.904	0.785	0.211	0	0.452
**S**	0.793	0.675	0.303	-0.011	0

The values of upper triangular matrices are the significance value (p-value). The values of lower triangular matrices for ANOSIM are R value.

Table S6 The dissimilarity analysis among different time groups

ANOSIM	OS	CK	30d	60d	90d
**OS**	0	0.002	0.001	0.001	0.001
**CK**	0.874	0	0.001	0.001	0.001
**30d**	0.835	0.918	0	0.001	0.002
**60d**	0.973	0.784	0.424	0	0.001
**90d**	1	0.952	0.907	0.647	0

The values of upper triangular matrices are the significance value (p-value). The values of lower triangular matrices for ANOSIM are R value.
